# Prevalence and Nonpharmacological Interventions for Sarcopenia among Cirrhotic Patients

**DOI:** 10.1155/2021/8866093

**Published:** 2021-02-04

**Authors:** Walid Kamal Abdelbasset, Gopal Nambi, Shereen H. Elsayed, Samah A. Moawd, Ahmed A. Ibrahim, Anju Verma, Sayed A. Tantawy, Dalia M. Kamel, Ayman K. Saleh, Osama R. Aldhafian, Naif Bin Nwihadh

**Affiliations:** ^1^Department of Health and Rehabilitation Sciences, College of Applied Medical Sciences, Prince Sattam bin Abdulaziz University, Alkharj, Saudi Arabia; ^2^Department of Physical Therapy, Kasr Al-Aini Hospital, Cairo University, Giza, Egypt; ^3^Department of Rehabilitation Sciences, Faculty of Health and Rehabilitation Sciences, Princess Nourah Bint Abdulrahman University, Riyadh, Saudi Arabia; ^4^Department of Physical Therapy for Cardiovascular/Respiratory Disorders and Geriatrics, Faculty of Physical Therapy, Cairo University, Giza, Egypt; ^5^Department of Physical Therapy, College of Applied Medical Sciences, Ha'il University, Hail, Saudi Arabia; ^6^Department of Physiotherapy, Centre of Radiation, Oncology and Nuclear Medicine, Cairo University, Giza, Egypt; ^7^Department of Physiotherapy for Integumentary Problems, Faculty of Physical Therapy, Deraya University, Menia, Egypt; ^8^Department of Physiotherapy for Women's Health, Faculty of Physical Therapy, Cairo University, Giza, Egypt; ^9^Department of Orthopedic Surgery, College of Medicine, Prince Sattam bin Abdulaziz University, Alkharj, Saudi Arabia; ^10^Department of Orthopedic, Faculty of Medicine for Girls, Al-Azhar University, Cairo, Egypt

## Abstract

Sarcopenia is the most common feature of hepatic cirrhosis characterized by progressive loss of muscle mass and function and increases permanently the mortality and morbidity rates among those patients. The incidence of sarcopenia in cirrhotic patients ranged 40-70% associating with impaired quality of life and augmented rates of infection. Based on these issues, this review is aimed at determining the prevalence and main causes of sarcopenia among cirrhotic patients and recognizing the recent diagnostic and physical treatment modalities that prevent risk factors for sarcopenia in those patients. No ideal modality is currently demonstrated for diagnosing sarcopenia in hepatic diseases, particularly cirrhosis; however, recent studies reported different diagnostic modalities for muscle function in different individuals including handgrip strength, skeletal muscle index, six-min walk test, liver frailty index, short physical performance battery, and radiological assessments for quadriceps and psoas muscles. Exercise training and therapeutic nutrition are strongly recommended for controlling sarcopenia in cirrhotic patients. The exercise program is designed and carried out on a frequent basis within an extensive scheduled time aimed at improving functional performance, aerobic capacity, and healthy conditions. Finally, a combination of exercise training and therapeutic nutrition is powerfully recommended to control sarcopenia in cirrhosis.

## 1. Introduction

Chronic hepatic diseases are a serious health problem worldwide. Annually, more than 1.2 million deaths occur as a result of hepatic cirrhosis [1]. A previous study provided that cirrhosis is the foremost widespread type of hepatic disease which accounts for the eighth common cause of death among individuals who underwent the public health treatment systems [2]. Cirrhosis is considered a primary cause of increased mortality rate worldwide and also related to a notable decrease in life quality [3]. The farthest treatment of a cirrhotic liver is transplantation [4, 5]. Expecting the development of the cirrhotic liver to promote curative decision becomes a serious challenge, particularly for cirrhotic individuals who may find a donor because of its exorbitant cost. Consequently, predictive factors that could be equipped to identify continued existence without transplantation are required [6]. Frequently, liver cirrhosis is associated with protein-energy malnutrition and low physical activity that may lead to sarcopenia [7, 8]. The incidence of protein malnutrition is ranged 20-30% of chronic hepatic disease patients and more than 60% of cirrhotic patients [7–9].

Sarcopenia is the most common feature of hepatic cirrhosis characterized by progressive loss of muscle mass and function and increases permanent mortality and morbidity rates among those patients [7, 9]. The incidence of sarcopenia in cirrhotic patients is ranged 40-70% associating with impaired quality of life and augmented rates of infection [10, 11]. Sarcopenia is more prevalent in cirrhosis than in other gastrointestinal diseases because of the distinctive disturbed metabolism induced by hepatic failure [12]. In keeping with the declaration of the European working group on sarcopenia in older people, sarcopenia is a syndrome combined with risky effects including a low level of physical activity, reduced quality of life, and deaths that distinguished with severe loss of skeletal muscle mass and function including muscle strength and performance [13].

The conventional status of sarcopenia was documented as a slow-developing age-related condition that is associated with a replacement of muscle fiber with fats, metabolic changes, oxidative stress, and muscle fibrosis in combination with deteriorations of the neuromuscular junctions. Orderly, it is known as a multifactorial condition in aging that takes place as a result of disturbances in physical activities, mitochondrial functions, hormonal secretions, and inflammatory mediators [13]. Despite the number of these disturbances that occur in cirrhotic patients, they are identified as particular factors that result in loss of muscle mass and function in those patients [14].

Although these characteristics, no specific criteria are demonstrated to diagnose this serious condition of hepatic cirrhosis, its poorly understood pathogenesis, and its treatment modalities that were not well investigated in the previous studies. For that, this review is aimed at summarizing the pathogenesis, diagnostic tools, and physical treatment modalities of sarcopenia in cirrhotic populations.

## 2. Causes of Sarcopenia in Cirrhosis

Different factors were documented that could result in sarcopenia in hepatic cirrhosis in spite of their exact role still not clarified. This status is commonly related to a disturbed balance between muscular anabolism and catabolism, although the particular mechanism included could be different between cirrhotic patients [14]. Exploring the roles of these critical factors may help to present evidence based on promoting approaches to manage and prevent muscle loss in cirrhotics and also to reduce mortality and morbidity related to sarcopenia.

The impaired muscular formation is combined with impaired energy intake as a result of impaired gastric residuals, nausea, and malabsorption of macronutrients induced by biliary dysfunction and portal hypertension [15]. Some circulating essential amino acids, particularly branched-chain amino acids (BCAAs) are decreased in hepatic cirrhosis because of their consumption in muscular fibers to release the raised serum ammonia through breaking down the glutamine, preventing muscles from its favorite fuel [16].

Serum insulin and testosterone are decreased in cirrhotic males, eliminating the stimulants of muscular formation and leading to myostatin upregulation which further prevents muscular formation [10, 15]. Consequently, elevated muscular breakdown in cirrhotic patients results from impaired liver glycogen storage, causing elevated consumption of lipids and breakdown of proteins [17]. It was reported that impaired mitochondrial function leads to muscular autophagy which is likely intermediated by elevated serum ammonia [18] and thereby activating the Ubiquitin-Proteasome Pathway (UPP), which could be associated with augmented systemic inflammatory markers that occur in hepatic cirrhosis [19]. Also, it was documented that energy demands are increased in severe cirrhotic patients when comparing them with matched healthy subjects [20], which could be due to the integration of elevated systemic inflammatory markers and loss of heat because of vasodilatation and demonstrated ascites, which provide further loss of muscular mass. Also, previous documents have completely discussed the predisposing risk factors [14, 18]. Unknown influences of many reasons for sarcopenia in cirrhotic patients have still remained. As a result of different contributing factors in their prognosis, it could be that each factor has a different reaction to therapeutic interventions of sarcopenia in cirrhotic patients.

## 3. Diagnostic Modalities of Sarcopenia in Cirrhosis

No ideal modality is currently demonstrated for diagnosing sarcopenia in hepatic diseases, particularly cirrhosis. Present modalities have a tendency to assess one form of sarcopenia specifically reduction in muscle mass or function of particular muscle groups. A small number of documents reported different evaluation methods for muscle function in different individuals including handgrip strength, skeletal muscle mass index, six-min walk test, liver frailty index, short physical performance battery, and radiological assessments for quadriceps and psoas muscles [21–30].

Handgrip strength is one of the functional assessments that are mostly used and well examined in sarcopenia in different individuals. Handgrip strength has been extensively assessed to investigate sarcopenia in cirrhotic individuals using a standardized digital dynamometer [21, 24, 31].

The skeletal muscle mass index has been used to assess sarcopenia using a bioelectrical impedance analysis that is identified as a noninvasive modality used to assess body composition through electrical signals across body tissues. This tool can determine fat mass by approximating entire body water and thereby safely estimates muscle mass in cirrhotic individuals [11, 21, 27]. Recently, the liver frailty index in combination with handgrip strength, balance, and chair standing has been used to predict the mortality rate in cirrhotics [28]. The short physical performance battery has been also used to assess gait speed and balance in older individuals in anticipation of liver transplantation [29]. Furthermore, the six-minute walk test has been used to assess the functional performance in individuals with cirrhosis through assessing the distance in meters walked during six minutes [30].

Dual-energy X-ray absorptiometry (DEXA) is commonly used to analyze body composition and distributes body contents into lean mass, fat mass, and bone mass. DEXA has been lately used to predict sarcopenia-related mortality in cirrhotic men [22]. Also, magnetic resonance imaging (MRI) has been identified as a likable assessment modality for examining muscle waste because of the low-dose exposure to radiation and high priority image involving well description of muscle mass by penetrating fats [25, 32].

Diagnostic ultrasound is the easiest and costless instrument that has been engaged in the assessment of muscle mass, mostly the quadriceps muscle that has been approved as a valid objective diagnostic modality for sarcopenia in candidates admitted in the intensive care unit [23, 33].

In addition, cross-sectional image is definitely the most common diagnostic tool of sarcopenia in cirrhotics that assess the skeletal muscle index on computed tomography (CT) at the L3 vertebrae level. Using abdominal CT scan at umbilical and L3 levels to assess the skeletal muscle mass index and psoas muscle thickness, it was found that sarcopenia of the psoas muscle is a predictor of mortality in cirrhotic patients [26]. The main modalities that were considered in the diagnosis of sarcopenia in cirrhotic patients are shown in Table 1.

In addition to assessments of muscle function, the subjective global assessment tool is regularly employed to assess the nutritional status of cirrhotic patients. This tool is usually used to assess the changes in weight, dietary intake, physical activity, and functional capacity. Cirrhotics are categorized as well-nutrition (Class A), mild to moderate malnutrition (Class B), and severe malnutrition (Class C) [34]. This subjective global assessment tool is commonly used in moderate and severe malnutrition in cirrhotic patients, approximately 50% [35] and increased depending on the severity of the condition [36].

## 4. Nonpharmacological Interventions for Sarcopenia in Cirrhosis

Exercise training and nutritional therapy are strongly recommended for controlling sarcopenia in cirrhotic patients. The major potential recommendation for exercise training in chronic diseases is low-starting, slowly increasing, and attentive to exercise symptoms [37]. The exercise program is designed and carried out on a frequent basis within an extensive schedule time aiming to improve functional performance, aerobic capacity, and healthy conditions [38]. The type of exercise, duration, frequency, and intensity should be demonstrated before starting the exercise program [39–42].

Currently, there are no available documents demonstrating the guidelines of exercise intervention in cirrhosis. While recent clinical studies demonstrated some information on this issue, the majority of these studies included cirrhotic patients with age 40-50 yrs, and most patients have conducted a particular nutrition therapy combined with exercise intervention. Basically, exercise training programs included cycling and walking exercises. The total duration of the exercise session ranged 30-60 minutes (warming up, exercise training, and cooling down), thrice a week in addition to home exercise. Based on the guidelines demonstrated for healthy individuals to prevent exercise-induced injuries, the exercise program is preferable to be classified into 3 phases or more in the early cirrhosis stage. Therefore, knowing the exercise training programs in noncirrhotic patients is strongly required [39].

Additionally, therapeutic nutrition is an imperative modality in the complementary intervention of cirrhotic patients. Many studies recommended the emerging of dietary intake and exercise training in this disease [15, 43–47]. The dietary program and required energy intake in the cirrhotic patients who conducted exercise training are established according to the cirrhosis stage and comorbidities, and also, the intensity of prescribed exercise should be considered to avoid catabolism [48]. Once the required energy intake is calculated, it should be classified between proteins, fats, and carbohydrates. The particular value of nutrients is commonly associated with various factors such as physical activities and other related comorbidities [7]. To distribute the required energy intake, the quantity of proteins is firstly determined in accordance with body weight and nutritional conditions of the patients [7]: 1.2 g/kg/day for normal nutrition, 1.3 g/kg/day for mild malnutrition, 1.4 g/kg/day for moderate malnutrition, and 1.5 g/kg/day for severe malnutrition. Secondly, the carbohydrates are determined as 45-65% of the total required energy. This percentage is differentiated from a patient to others; 55% is the minimum required carbohydrate for the patients who conduct exercise training. Accordingly, fats should be the remaining calorie intake. Cirrhotic patients are recommended to ingest 1 ml minimally per 1 kcal in their diet and also should maintain sufficient hydration before and during exercise training [49]. The main intervention modalities that were considered in the treatment of sarcopenia in cirrhotic patients are described in Table 2.

## 5. Conclusions and Recommendations

Sarcopenia is more prevalent in cirrhotic patients than in other gastrointestinal diseases because of the distinctive disturbed metabolism induced by hepatic failure. Sarcopenia is associated with impaired energy intake and low physical activity levels. No ideal modality is currently demonstrated for diagnosing sarcopenia in hepatic diseases, particularly cirrhosis; however, recent studies reported different diagnostic modalities for muscle function in different individuals including handgrip strength, skeletal muscle index, six-min walk test, liver frailty index, short physical performance battery, and radiological assessments for quadriceps and psoas muscles. Exercise training and therapeutic nutrition are strongly recommended for controlling sarcopenia in cirrhotic patients. The exercise program is designed and carried out on a frequent basis within an extensive schedule time aimed at improving functional performance, aerobic capacity, and healthy conditions. Finally, the combination of exercise training and therapeutic nutrition is powerfully recommended to control sarcopenia in cirrhosis.

## Figures and Tables

**Table 1:** Main diagnostic modalities of sarcopenia that were considered in cirrhotic patients.

Author (year)	Modality of diagnosis	Cut-off point for sarcopenia in cirrhotics
Saeki et al. (2019) [21]	Skeletal muscle mass index (kg/m^2^) using bioelectrical impedance analysis and handgrip strength (kg) using a digital dynamometer based on the criteria of Asian working group for sarcopenia (AWGS), Japan society of hepatology (JSH), and European working group on sarcopenia in older people (EWGSOP2).	For skeletal ms mass index: males ≤ 6.80 kg/m^2^ and females ≤ 5.43 kg/m^2^. For handgrip strength: males ≤ 24.2 kg and females ≤ 15.8 kg
Sinclair et al. (2019) [22]	Dual-energy X-ray absorptiometry (DEXA) for upper limb lean mass/height-adjusted (kg/m^2^).	Males < 1.6 kg/m^2^
Hari et al. (2019) [23]	Diagnostic ultrasound for quadriceps and psoas muscle diameter to height ratio (cm).	Not detected. While the psoas diameter to height ratio was strongly correlated with the mortality rate of cirrhotic patients
Hanai et al. (2019) [24]	Hand-grip strength (kg).	Males < 30 kg and females < 15 kg
Praktiknjo et al. (2018) [25]	Magnetic resonance imaging (MRI) at the level of superior mesenteric artery on the fat-free muscle area (mm^2^).	Males < 3197 mm^2^ and females < 2895 mm^2^
Gu et al. (2018) [26]	Computed tomography (CT) for psoas muscle thickness adjusted/height (mm/m) and area (mm^2^).	For thickness/height: males < 17.3 mm/m and females < 10.5 mm/m. For muscle area: males < 1561 mm^2^ and females < 1464 mm^2^
Belarmino et al. (2017) [27]	Phase angle in degrees through bioelectrical impedance analysis of the skeletal muscle index (kg/m^2^).	Phase angle ≤ 4.9°, skeletal muscle index < 7 kg/m^2^ for Males < 5.7 kg/m^2^ for females
Lai et al. (2017) [28]	Liver frailty index, depending on gender, handgrip strength, and balance with a ranged score of 0-7.	Frailty ≤ 4.5
Wang et al. (2015) [29]	Short physical performance battery assessing frailty with a score up to 12.	Frailty ≤ 9
Carey et al. (2010) [30]	Six-min walk test (m).	Sarcopenia with <250 m

**Table 2:** Main intervention modalities that were considered in the treatment of sarcopenia in cirrhotic patients.

Author (year)	Modality of treatment	Outcome measures	Main findings
Kruger et al. (2018) [43]	Nutrition plus home exercise.	Six-min walk test and VO_2_ peak.	↑ 6MWT and VO_2_ peak (*p* < 0.05).
Berzigotti et al. (2017) [47]	Nutrition plus 40 min of aerobic and strengthening exercises.	Weight and hepatic venous pressure gradient.	↓ Hepatic venous pressure and body weight (*p* < 0.05).
Román et al. (2016) [40]	Relaxation plus 10-30 min of moderate-intensity cycling and treadmill walking exercise.	Ventilatory anaerobic threshold, effort time, body fat, and Up & Go test.	↑ Ventilatory anaerobic threshold, effort time. ↓ Body fat and Up & Go test.
Macías-Rodríguez et al. (2016) [44]	Nutrition plus 40 min cycling and kinesiotherapy.	Ventilator threshold and ventilatory efficiency.	↓ Ventilator threshold and ↑ ventilatory efficiency and physical activity (*p* < 0.05).
Debette-Gratien et al. (2015) [41]	Therapeutic educations with 20 min cycling exercise.	Six-min walk test, VO_2_ peak, muscle strength, ventilator threshold.	↑ VO_2_ peak, muscle strength, 6MWT, and ventilatory threshold (*p* < 0.05).
Román et al. (2014) [45]	10 gm of leucine/day plus cycling, treadmill walking, and resisted exercises.	Exercise capacity, muscle mass, and quality of life.	↑ Exercise capacity, muscle mass, and quality of life (*p* < 0.05).
Zenith et al. (2014) [42]	30 min moderate intensity of cycling aerobic exercise.	VO_2_ peak, muscle mass, and quality of life.	↑ VO_2_ peak, muscle mass, and quality of life (*p* < 0.01).
Pattullo et al. (2013) [46]	Nutrition plus walking for 10,000 steps.	Steps per day, dietary intake, BMI, and HOMA-IR.	↑ Steps per day, and ↓ dietary intake, BMI, and HOMA-IR (*p* < 0.05).

## Data Availability

No data were used to support this study.
